# A Comprehensive Review of the Coffee Leaf Miner *Leucoptera coffeella* (Lepidoptera: Lyonetiidae)—A Major Pest for the Coffee Crop in Brazil and Others Neotropical Countries

**DOI:** 10.3390/insects12121130

**Published:** 2021-12-17

**Authors:** Juliana Dantas, Isabela O. Motta, Leonardo A. Vidal, Eliza F. M. B. Nascimento, João Bilio, Júlia M. Pupe, Adriano Veiga, Carlos Carvalho, Rogerio B. Lopes, Thales L. Rocha, Luciano P. Silva, José R. Pujol-Luz, Érika V. S. Albuquerque

**Affiliations:** 1 Embrapa Genetic Resources & Biotechnology, Brasília 70770-917, Brazil; juliana.dantas@embrapa.br (J.D.); isabela.motta94@gmail.com (I.O.M.); leonardoamorimvidal@gmail.com (L.A.V.); lizabellard@gmail.com (E.F.M.B.N.); j_525@hotmail.com (J.B.); juliampupe@gmail.com (J.M.P.); rogerio.lopes@embrapa.br (R.B.L.); thales.rocha@embrapa.br (T.L.R.); luciano.paulino@embrapa.br (L.P.S.); 2 Embrapa Cerrados, Center for Plant Production Systems (SPV), Planaltina 73310-970, Brazil; adriano.veiga@embrapa.br; 3 Embrapa Coffee, Alameda do Café, 1000, Varginha 37026-400, Brazil; carlos.carvalho@embrapa.br; 4 Department of Zoology, Institute of Biological Sciences, University of Brasilia, Brasília 70910-900, Brazil; jrpujol@unb.br

**Keywords:** resistance, cultivar, biopesticide, biological control, chemical control, life cycle, CLM

## Abstract

**Simple Summary:**

Coffee is produced in more than 60 countries by 25 million coffee producers, most of whom are smallholders in emergent countries. More than a beverage intake, coffee has become a ritual for an increasing number of consumers across the globe. This rising market demands modern management to improve grain quality, environment protecting, and produce pesticide-free products. Among several challenges to overcome, the coffee leaf miner (CLM) pest is one of the most severe threats to the coffee crop, especially in neotropical countries, such as Brazil, the world’s largest producer. Responsible for losses ranging from 30–70%, the CLM impairs the grain production and quality, which negatively reflects on the coffee production chain. Drawback aspects caused by chemical control with pesticides, such as the harmful effects to human health and environment and the selection of resistant-insect populations, prompt scientists to improve integrated pest management (IPM) tools. Therefore, the development of new resistant cultivars, biological control, nano-biopesticide products and other approaches are important strategies to a sustainable CLM control design. This review addresses basic knowledge of the insect *L. coffeella* and proposes novel insights for an IPM view.

**Abstract:**

The coffee leaf miner (CLM) *Leucoptera coffeella* moth is a major threat to coffee production. Insect damage is related to the feeding behavior of the larvae on the leaf. During the immature life stages, the insect feeds in the mesophyll triggering necrosis and causing loss of photosynthetic capacity, defoliation and significant yield loss to coffee crops. Chemical control is used to support the coffee production chain, though market requirements move toward conscious consumption claiming for more sustainable methods. In this overview, we discuss aspects about the CLM concerning biology, history, geographical distribution, economic impacts, and the most relevant control strategies in progress. Insights to develop an integrated approach for a safer and eco-friendly control of the CLM are discussed here, including bio-extracts, nanotechnology, pheromones, and tolerant cultivars.

## 1. Introduction

Since the VI century, coffee consumption has expanded from goats to human beings and has been increasingly gaining followers. Its stakeholders operate not only in planting, harvesting, roasting, packaging, transporting, and blending, but also in wholesale and retail marketing ranging from modest diners to sophisticated restaurants and high-profile tasting contests. Although coffee is a non-essential food commodity, its commercial chain is one of the most profitable and complex in the world with the 2020/21 yield estimated at 176.1 million bags [1], produced in several countries, such as Brazil, Vietnam, Colombia, Indonesia among others [1,2]. Brazil is currently the largest producer and exporter, with a record expectation of 67 million bags of processed coffee [1], where few states account for more than 90% of national production [3].

The challenges for maintaining this productive chain are many, among which are the pests that threaten the crop, including some of the phytophagous organisms as leaf miner *Leucoptera coffeella* (Guérin-Mèneville and Perrottet) (Lepidoptera: Lyonetiidae), a coffee exclusive enemy [4], the coffee berry borer *Hypothenemus hampei* (Coleoptera: Scolitidae), several species of cochineal (coccids, pseudococcids, and diaspidids) and cicadas (Hemiptera), and mites (Acari).

The coffee leaf miner (CLM) (*L. coffeella*), is considered one of the most important coffee pests due to the high damage this moth causes to coffee plantations. The damage is a result of injuries caused by its larvae that feed on the palisade parenchyma of the coffee leaves, which consequently reduces fruit production [5]. Estimated losses in neotropical producing countries can reach up to 87% of the coffee productivity, depending on the season, the defoliation can reach up to 75% [6,7]. High infestation rates of *L. coffeella* can cause losses above 50% in Brazil and Colombia [8,9,10,11], vary from 20% to 40% in Puerto Rico and around 12% in Mexico [12,13]. The CLM incidence surveys are flawed because the monitoring is done by sampling mined leaves or traps and this system is not ideal. New systems employing aerial images and terrestrial photogrammetry are being developed to facilitate its detection in the field [14,15].

Consumer expansion in new markets has led coffee growers to search for sustainable production systems, like adoption of lower environmental impact agricultural practices leading to greater economic value of the product [16,17,18]. The selection of resistant populations has been reported for most of the insecticides currently used in preventive approaches [11]. Although important as a natural mortality factor, biological control agents are not efficient when used as a stand-alone strategy. Therefore, biotechnological strategies can generate alternatives to meet the demand for sustainable, durable, and safe solutions for specific CLM control. In addition, currently, to access more demanding markets, farmers must use methods consistent with the integrated pest management (IPM) philosophy, and not just chemical control methods [19,20,21].

## 2. History, Origin, and Distribution

Despite its origin in the African continent, CLM was first reported 178 years ago in coffee plantations in the Caribbean Antilles [22,23,24]. It was first named *Elachista coffeella*, then assigned to *Bucculatrix* sp. (Stainton, 1858) and later in *Cemiostoma* sp. (Stainton, 1861). Finally, it was included in the genus *Leucoptera* (Meyrick, 1895) and named *L. coffeella* in 1897 by Lord Walsingham. It was already reported as *Perileucoptera coffeella*, synonymous [25].

**Figure 1:**
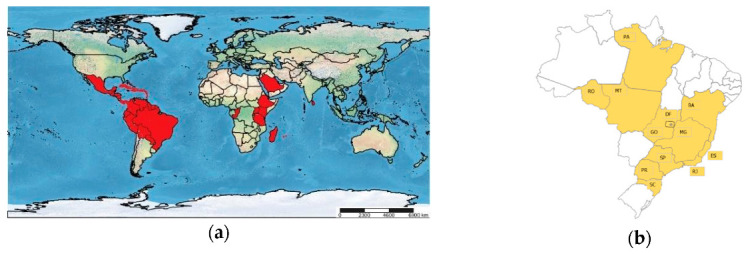
Presence of *L. coffeella* in: (**a**) The world map, showing producing countries highlighted in red: North and Central America: Antigua and Barbuda, Barbados, Costa Rica, Cuba, Dominica, Dominican Republic, El Salvador, Grenada, Guadeloupe, Guatemala, Haiti, Honduras, Jamaica, Martinique, Mexico, Montserrat, Nicaragua, Puerto Rico, Saint Lucia, Saint Vincent and Grenadines, Trinidad and Tobago; South America: Bolivia, Brazil, Colombia, Ecuador, French Guiana, Guyana, Peru, Suriname and Venezuela; Africa: Reunion, Mauritius, Madagascar, Uganda, Kenya, Congo, Ethiopia, Tanzania and Rwanda; Asia: Saudi Arabia and Sri Lanka, and (**b**) Brazil, highlighting in yellow the affected producing states: RO = Rondônia, MT = Mato Grosso, PA = Pará, GO = Goiás, DF = Distrito Federal, BA = Bahia, MG = Minas Gerais, ES = Espírito Santo, SP = São Paulo, RJ = Rio de Janeiro, PR = Paraná, SC = Santa Catarina.

*L. coffeella* is now a cosmopolitan pest (Figure 1a) and occurs in the leaves of coffee plants in Africa, Asia, and Neotropical countries, comprising Central America, the Caribbean islands, and South America [23,26,27,28]. In Brazil, the presence of CLM was detected around the 19th century and became a key pest of coffee culture in the country [29]. Since then, wherever coffee is grown, the CLM is present [30,31,32] (Figure 1b) specially in the Brazilian Savanna Biome called Cerrado.

## 3. Biology

### 3.1. Life Cycle

**Figure 2:**
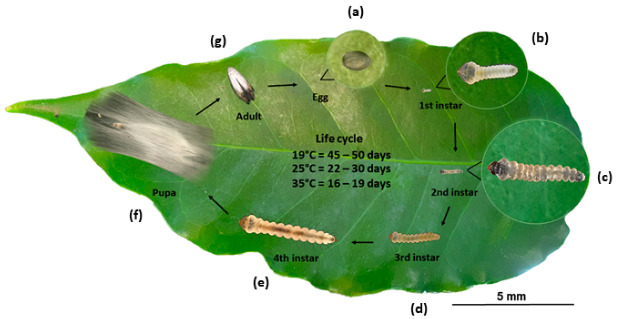
*L. coffeella* life stages from egg to adult. After hatching the egg, (**a**) the larvae development is divided into four instars: L1 (**b**), L2 (**c**), L3 (**d**), and L4 (**e**). The last instar forms a cocoon and turns into pupa (**f**). The adult emerges (**g**) from the pupa to mate. Eggs are laid over the adaxial side of the coffee leaf and the cycle restarts. Temperature rising accelerates and shortens the cycle span time, as detailed.

The CLM is a holometabolous insect, its life cycle includes different stages [33,34] (Figure 2). Considering a 25 °C temperature, the egg stage usually lasts about five days, the larval stage lasts about twelve days, and the pupae lasts about five days, totaling approximately 22 days until reaching adulthood [35]. Total life cycle varies according to temperature, relative humidity, and rainfall. In the dry season, the attack of the pest is generally more severe than in wet periods [36,37].

**Figure 3:**
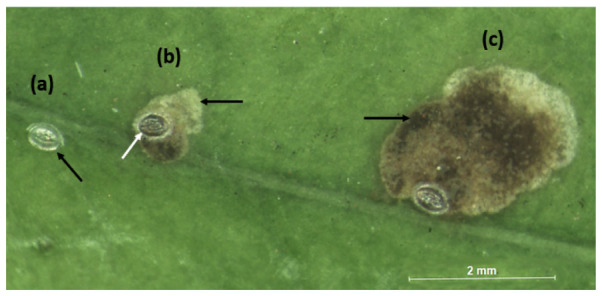
*L. coffeella* egg hatching and mine progression: (**a**) Unhatched eggs have a translucent structure, the arrow indicates a freshly oviposited egg; (**b**) after hatching, the eggshell becomes darker (white arrow) and the larva penetrates the leaf under the egg and starts feeding, forming a light green mine (black arrow); (**c**) enlargement of the mine. The black arrow indicates the dark color of the mine due to residues left behind by the larva.

The egg is about 0.3 mm, made by a translucent structure, with an oval, concave shape, and expanded sides [23,36]. After hatching, the larvae leave the underside of the eggs, which are in contact with the upper leaf epidermis, and get into the leaves [38] (Figure 3).

**Figure 4:**
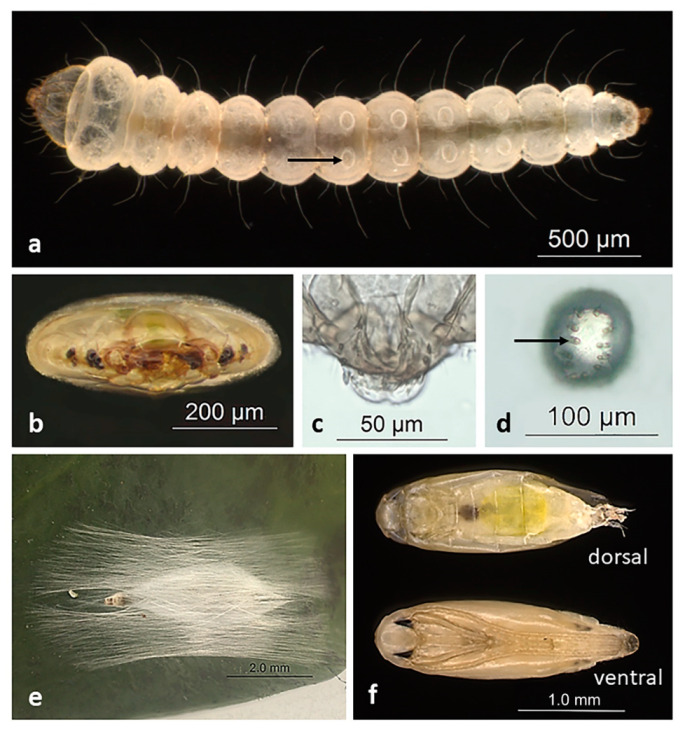
Immature stages of *L. coffeella*. (**a**) Ventral view of a fourth instar larva. Arrow indicates proleg; (**b**) flat head in front view; (**c**) chewing mouthpiece; (**d**) crochets (arrow) located in proleg. Morphology details to distinguish the immature stages are described in the text; (**e**) pupa’s sea cocoon; (**f**) pupae’s dorsal (**a**), and ventral (**b**) shapes.

The *L. coffeella* larval phase has four instars [34,39]. Newly hatched larvae have a translucent whitish color, but throughout their development they take on a greenish yellow tone. The last larval instar is about 4–5 mm, flattened, segmented with 11 segments, and yellowish in color [22,23] (Figure 4a). Fourth instar larvae have a flat head and mouthpiece of the chewing type (Figure 4b,c), prolegs, and crochets [23,34,40] (Figure 4d).

After accomplishing the larval stages, the larvae leave the mines and weave a silk X-shaped cocoon, usually in the axial region of the leaf, forming the pupae [23,36] (Figure 4e). Pupae have an approximate length of 2 mm, milky color, small black eyes, antennas, and legs ventrally fused, and wrinkled wings [23] (Figure 4f). Usually, more pupae are found in the “skirt” region of coffee plants, which is the underside of the plant where dead leaves accumulate [38].

**Figure 5:**
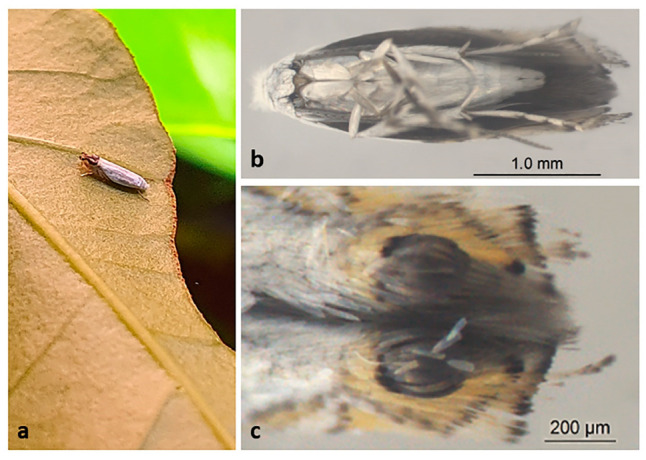
*L. coffeella* adults (**a**) perched on coffee leaf; (**b**) male seen by ventral view, with white scales all over the body; (**c**) closed caption of the wings apex from dorsal view to show details: black circle surrounded by yellow bristles.

From the pupae, adults emerge with an average body length of 2 mm and a wingspan of 6.5 mm (Figure 5a). They have a head with “white hair scales”, long antennae that reach the end of the abdomen, silver white chest, legs covered with white bristles, wing with three rows of yellow bristles at the apex with a black circle, yellowish abdomen and covered with white scales and genital organs covered by a tuft of white scales [22,23] (Figure 5b,c). A recent description of the sexual polymorphism [34] shows the differentiation of the structures present in both male and female genitalia: male—bulbus ejaculatorius, valve, gnathos, and aedeagus and female—ovipositor, sclerite, and corpus bursae. Overall, the female whole body is similar to the male, except by a slightly longer average length.

### 3.2. Larval Feeding Behavior

**Figure 6:**
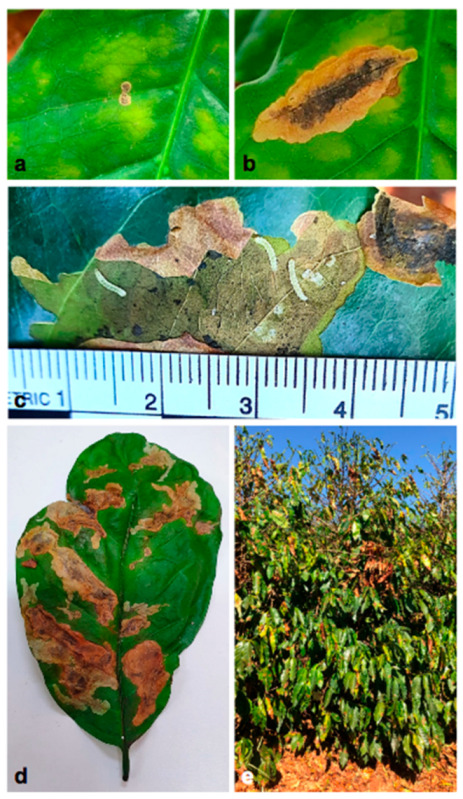
Damage to coffee leaf caused by the CLM larvae. (**a**) Initial mine formation; (**b**) mine developed, with large necrotic area; (**c**) larvae inside the mine; (**d**) leaf with impaired photosynthetic surface; (**e**) coffee defoliation.

CLM is a monophagous pest, coffee exclusive and the larvae are the causal agent of the crop damage [4]. When feeding on the mesophyll of the coffee tree leaves, the insect creates mines that justify the common name of the pest—coffee leaf miner (Figure 6a). The mines cause necrosis (Figure 6b) and reduce the photosynthetic leaf surface (Figure 6c,d), leading to a lower photosynthetic rate of the plants and consequent depletion of the plant and productivity diminish [33]. The damage caused by this insect includes defoliation [36] (Figure 6e). Eventually, without adequate cultural treatments, the infestation can lead to the death of the plant.

A relationship between the feeding damage of CLM and the application of synthetic fertilizers has been described in the literature [41,42]. The amount of free amino acids and reducing sugars in the metabolic system of coffee plants is related to nutritional imbalance and susceptibility to pests. Plants fertilized with organic material showed a decrease of up to 50% of leaf mines [41].

### 3.3. Adult Behavior

*L. coffeella* is depicted as the quintessence of sensitivity level [37]. In adulthood, the insect has a nocturnal habit and during the day, it shelters beneath the coffee leaves [36]. Mating and laying occur preferably at night [38,39,43]. The sexual behavior of adults is very peculiar and can present the following stages: (1) Females remain in a resting position with the abdomen curved downwards, exposing the pheromone gland in continuous movements from the inside out, to attract males; (2) when perceiving the pheromone, males remain in the same place, moving their antennae and flapping their wings, and then walk toward the female; (3) male touches the female with his antennae, female retracts the pheromone gland and places the abdomen toward the male; (4) the male places his abdomen toward the female abdomen, releases the aedeagus and fits the female, initiating copulation [44]. Females usually oviposit in the upper epidermis of the leaves at nightfall [36,38].

## 4. Losses Caused by the CLM

Brazil, Vietnam, and Colombia are responsible for about 50% of the global production of coffee [1]. The Brazilian coffee commodity accounts for more than one-third of world coffee production and exports, figuring in the 5th place in Brazilian exportation agricultural trades [45,46] performing US$ 1.3 billion in 2020 [47].

**Figure 7:**
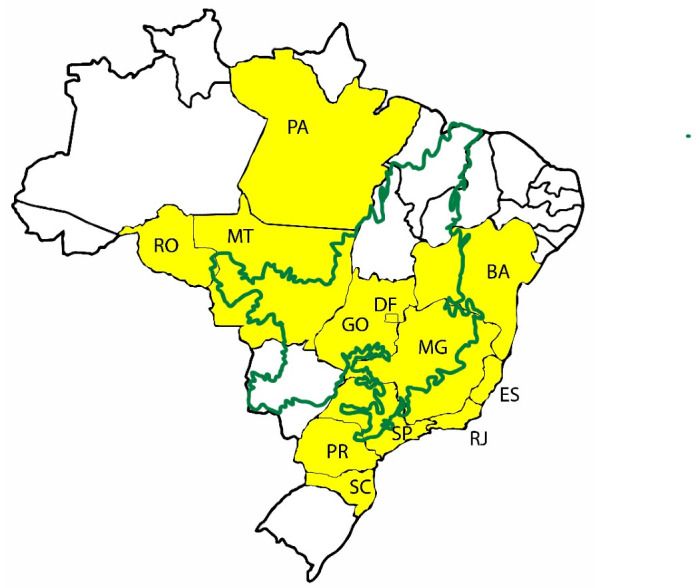
Map of the Brazilian territory indicating the coffee producing states (yellow) and the Cerrado biome delimited in green.

The intensity of the damage caused by larvae of CLM is associated to a series of factors, such as cultivars, crop management, climate, altitude, season, indiscriminate use of phytosanitary products, and presence of adventitious and associated plants, among others [2,48,49]. Until 1970 CLM outbreaks in coffee plantations in Brazil were sporadic because of the effective action of natural enemies on the CLM population. Moreover, coffee crops used to be organized with narrow spacing, which is an adverse condition for this pest. In the 1970s, the mechanized model replaced the former one, requiring large areas of extensive agriculture with greater spacing between trees. The new plantation areas expanded onto drier and warmer regions, such as Cerrado [33], where the main producing states are currently located (Figure 7).

Cerrado covers more than 200 million hectares, equivalent to about 23% of the national territory, distributed in the States of Minas Gerais, Goiás, Mato Grosso, Mato Grosso do Sul, Tocantins, Bahia, Piauí, Maranhão, and the Federal District [50]. It is characterized by having two well-defined seasons (dry winter and rainy summer), where the dry period varies from four to seven months and rains are concentrated from October to March [51], with an average temperature around 22 to 27 °C. For these reasons, coffee production in Cerrado is favored and occurs significantly.

However, Cerrado climatic conditions also favor the CLM, which during their normal life cycle females are able to oviposit around seven eggs per night and more than 50 eggs during their lifetime [36]. High temperature in coffee fields often allows two CLM rounds in producer regions [35]. On Cerrado conditions, CLM can reach 12 cycles per year, when considering the fact that the dry period in Cerrado can last seven months, and the life cycle can reach 16 days on hot periods. In a few days, the injuries area evolves from some millimeters to several centimeters (Figure 5) ending up to the falling of the leaves and lowering the productivity.

## 5. Control and Management of the CLM

Ideally, the first generation of the CLM must be controlled efficiently to prevent a growing population throughout the year. Accurate models, like a new system employing aerial images and terrestrial photogrammetry are being developed to facilitate its detection and estimate levels of infestation in the field [14,15,52].

The hot climate, typical of neotropical countries like Brazil, shortens the pest cycle, resulting in remarkably high populations of adults, larvae, and pupae, in addition to a large number of eggs in leaves [53]. On average, 8 generations can occur per year in Brazil, even reaching 12 during the crop year [54]. In addition to the current challenges with the pest, climate change scenarios predict an increased infestation of CLM due to a greater number of generations per month [30,55]. In this case, the coffee production would be highly threatened by increasing temperature and erratic rainfall that has taken place, reducing coffee yield by affecting growth and development of the plant, mostly by increased incidence of CLM [56].

Chemical control leads to selection of CLM populations resistant to most insecticides currently used in preventive approaches, in addition to presenting environmental and health restrictions [16]. In turn, biological control, although important as a natural mortality factor, are not efficient when used as a stand-alone strategy. In this scenario, the early control of CLM in sustainable production systems, such as bioproducts enhanced in association with nanotechnology, are more advantageous, as they use agricultural practices with less environmental impact, in accordance with the IPM, also resulting in greater economic value of the product [16,17,18,19]. 

### 5.1. Chemical Control

Chemical control is a tool of great importance for agriculture, needed to reduce populations of pest species that reach infestation levels able of causing economic damage, such as the CLM, which drastically reduces the productivity, mainly in cultivars of *C. arabica* and *C. canephora*. Currently, chemical control is considered the traditional way to combat CLM, however it has been reported as ineffective in the short and medium term due to the insect’s acquired resistance to most insecticides in use, requiring several applications, which increases costs and risks to the producer and the environment [57,58].

Classically, there are three mechanisms by which insects develop resistance to insecticides: (1) reduction of insecticide penetration through the insect’s cuticle, (2) detoxification or metabolism of the insecticide by enzymes, and (3) reduced sensitivity at the site of action of the insecticide by the nervous system [59]. Resistance to insecticides occurs by the selection of resistant individuals. Their reproductive advantages lead to increasing less-sensitive populations. To mitigate selection pressure caused by the overuse or misuse of pesticides, the Insecticide Resistance Action Committee (IRAC) management programs recommend insecticide applications in a rotation system composed of multiple effective modes of action. The use of different application methods (leaf-based or soil-based) with the same active ingredient must be avoided because this practice contributes to the emergence of resistant progenies [60].

**Figure 8:**
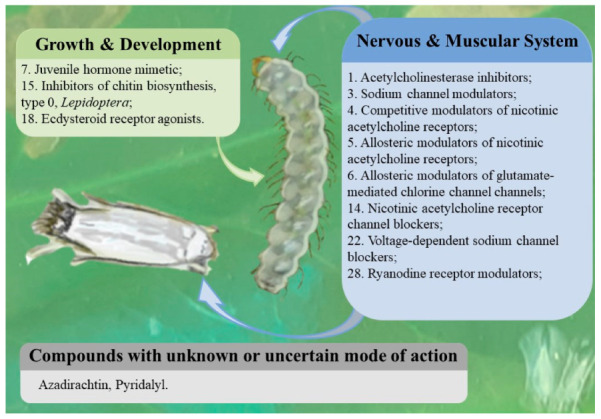
Main chemical products to control the CLM registered at the Brazilian Ministry of Agriculture, Livestock and Supply (MAPA), to control *L. coffeella*, (Supplementary Table S1) categorized by their action mode in squares with different colors: green—insecticides that affect the growth and the development; blue—insecticides that affect the nervous and the muscular system; grey—action modes not determined. The action mode is categorized by numbers, showing the specific action site of the product.

Insecticides are classified according to the main group or primary site of action, then by chemical subgroup or active ingredient [61]. The action of insecticides over Lepidoptera can be on the nervous and muscular systems, on the digestive system, on cellular respiration, on growth and development, or in an unknown way [62]. Currently, neurotoxic insecticides are the most used by coffee growers, such as organophosphates, carbamates, pyrethroids, and neonicotinoids (thiamethoxam) and diamides (chlorantraniliprole) [63], among the active compounds currently available and registered at the Brazilian Ministry of Agriculture, Livestock and Supply (MAPA) (Supplementary Table S1). The most used chemical compounds are aimed to the nervous system (Figure 8): 4-thiamethoxam [7], 28-chlorantraniliprole [11,63,64,65], and 14-cartap hydrochloride [66].

A nice performance in CLM control was observed using thiamethoxam in soil application, which resulted in both systemic action and specific results. Moreover, thiamethoxam gives protection above 180 days whereas control plants without insecticide showed a drastic decrease of more than 50% in production [7,67]. In Brazil, resistance to organophosphates in *L. coffeella* populations has been reported for a few decades [63,64,68,69]. However, resistance to thiamethoxam and chlorantraniliprole was recently detected in different coffee-producing states [11,63,65]. Similarly, resistance using chlorantraniliprole diamide was detected in other lepidopteran-pest populations, as *Plutella xylostella* [70,71], *Tuta absoluta* [72,73], and *Spodoptera frugipera* [74,75]. Other molecules that interfere in the growth and development or have unknown action specifically affecting CLM are depicted in Figure 8.

### 5.2. Genetic Resistance

Coffee farming began in Brazil in 1727, with the Typica cultivar introduction (*C. arabica*). This genetic material was nearly the only one exploited in a commercial basis until the middle of the 19th century [76]. Since then, several traits of interest, such as high yield and improved tolerance to biotic and abiotic factors, have been introgressed through genetic breeding generating several new cultivars [77,78,79]. Those genetic breeding programs allowed the expansion of coffee growing in several biomes in the country. However, traditional cultivars such as Catuaí and Mundo Novo, and even other cultivars with higher levels of tolerance to other pests, suffer heavy CLM infestation.

Regarding susceptibility to CLM, the coffee species have been classified as: highly resistant (*C. stenophyila*, *C. brevipes*, *C. liberica* and *C. salvatrix*); moderately resistant (*C. racemosa*, *C. kapakata*, *C. dewevrei,* and *C. eugenioides*) and susceptible (*C. congenesis*, *C. canephora,* and *C. arabica* [80]. However, coffee breeding programs deal with serious limitations to perform interspecific hybridization of *C. arabica* because of its low genetic variability and allotetraploidy [76].

The main source of resistance to CLM are plants derived from a natural cross between *C. arabica* (susceptible) and *C. racemosa* (moderately resistant) carried out in the 1950s [81]. Subsequently, individuals belonging to the second generation of natural backcrosses (RC2) with *C. arabica* [29] were hybridized with commercial cultivars aiming at the development of cultivars resistant to the CLM [77]. However, the inheritance of these traits remains unclear, which hinders its fixation in plants with high productivity and good quality grains. Currently, there are only two resistant cultivars to CLM in Brazil: Siriema VC4 [78] and Siriema AS1 [79]. Data based on the influence of chemical composition and leaf age of resistant genotypes suggest that young leaves are more efficient in controlling CLM than older leaves, with significant reduction in egg laying and increased larvae mortality, probably due to the higher concentration of secondary metabolites, such as phenols [38]. However, CLM tolerance is also observed in cultivars derived from the Sarchimor group, such as Obatã IAC 1669-20 and Tupi IAC 1669-33. A comparative study found that despite the higher percentage of leaves injured by the CLM, these cultivars were able to keep the leaves longer showing a better response in the face of CLM attack [43]. In addition, it was noticed that the destruction of the leaves is milder in *C. canephora* than in *C. arabica*. On the other hand, resistance of CLM may be of biochemical nature [29] in which the larvae probably have a protective mechanism against a possible toxic effect of caffeine [82].

The biotechnology achievements on genetic improvement of coffee plants were revised by Mishra & Slater (2012) [83], and more recently by Villalta-Villalobos & Gatica-Arias (2019) [84], showing that all the widely cultivated coffee species, *C. arabica*, *C. canephora,* and *C. liberica,* present limitations for their genetic improvement through conventional programs [85]. In this context, genetic engineering techniques are feasible alternatives to solve this barrier, and significant advances have been generated during the past decades [84]. Genetic engineering techniques were developed in *C. canephora* [86] and *C. arabica* [87] to express resistance-genes aiming to control coffee insect-pests. Transgenic plants carrying the *cry1Ac* gene from *Bacillus thuringiensis* showed good resistance to *L. coffeella* in greenhouse conditions and initial field defiance experiments [88]. Nonetheless, the constitutive promoter *EF1α-A1* provided too low Cry1Ac protein levels in the transgenic leaves to confer efficient and sustainable protection against *L. coffeella* in the field [89].

One promising technology that could be adopted in non-conventional plant breeding programs is the CRISPR/Cas9 genome editing technique, which allows the precise cut of a DNA molecule, managing to modify the genome sequence through elimination or insertion of new DNA [90]. The use of this technology in coffee was recently reviewed [84], showing that genome editing by CRISPR/Cas9 is an efficient and reliable way to inactivate genes of agronomic interest and that, in the near future, can be widely used in this culture [91].

### 5.3. Biological Control

Population dynamics of CLM can be strongly affected by host-plant attributes and environmental conditions, but also by the abundance of natural enemies [28,92,93]. Parasitoids and predators (wasps and ants) have been extensively reported in coffee plantations in several Latin American and African countries since 1970s as natural mortality factors [10,94,95,96,97,98,99]. Despite the great number of hymenopteran species parasitizing *Leucoptera* sp. larvae in coffee growing areas worldwide (Table A1) and their contribution on population dynamics of the pest, no significant attempt was made to use these natural enemies as biological control. As reviewed by [100], few cases of introduction of new parasitoids species or augmentation of indigenous species have been made for the suppression of CLM populations. Although unsuccessful in most of the cases, the author stressed the great potential of periodic releases of natural enemies of *L. coffeella* under certain conditions. Some invertebrate-pathogenic fungi were already tested against different developmental stages of species in the *Leucoptera* genus. Eggs and larvae of *L. coffeella* were susceptible to infection by the fungus *Metarhizium anisopliae* [101]. Moreover, the species *Beauveria bassiana* was described as pathogenic to *L. malifoliella*, infecting last instar larvae when leaving the leaf mines for pupation after exposure to this pathogen [102].

Crop management practices and landscape structure can affect insect communities and the ecosystem services provided by natural enemies, enhancing their diversity and abundance. Ecologically complex coffee systems are associated with higher biodiversity of parasitoid wasps, ants, and other predators [103]. For instance, richness and abundance of social wasps is positively correlated with forest cover in coffee-producing regions, increasing *L. coffeella* predation [104].

### 5.4. Semiochemicals

Semiochemicals are chemicals that mediate the interaction between organisms [105] within the same species (pheromones) or from different species (allelochemicals) [106]. Pheromones are able to attract or repel insects enhancing or inhibiting the action of other chemicals. They can be useful in biological control to manipulate or disrupt the natural behaviors of insects to reduce population levels and, consequently, decrease crop damage. Semiochemicals have the potential to be used in direct mass control of pests as a good alternative over huge plantations and, trapping or mating disruption, or in deterring pests from food and oviposition sites [107].

The main and secondary components of *L. coffeella* sexual pheromones are 5,9-di-methylpentadecane and 5,9-di-methylhexadecane, respectively [108,109]. The female production pattern of 5,9-di-methylpentadecane is related to the period of the day and the time after adult emerging from pupa [110]. The results concerning virgin females showed that the higher amounts of pheromone were produced in the period between 4 h before and 2 h after dawn and in 1 day-aged females. Host plants release volatiles which influence the mating and oviposition of lepidopterans and may increase biosynthesis of sexual pheromone. In the case of *L. coffeella*, the volatiles released by *C. arabica* increase the mating ratio by 90%. They accelerate the onset of copulation and soar mating duration. Moreover, the CLM oviposition observed in *C. arabica* is higher than in non-host plants [111].

Recent progress using “attract and kill” approaches have been reported for lepidopteran pests in the fields [112]. The monitoring with 5,9-di-methylpentadecane is indicated for the integrated management of the CLM. Delta trap monitoring can be done by installing one trap for every 4 hectares [113]. Traps containing synthetic sex hormone can be used 0.5 m above the ground for that the peak of male capture occurs around noon, coinciding with the peak mating time [44].

Mating disruption (MD) techniques provide prevention of mate location and mating, and factors that interfere with or delay the normal insect mating processes. Despite some successful cases reported, mating disruption treatments are still to be improved for CLM application [107,114]. The viability of MD to reduce coffee leaf miner populations by application of synthetic 5,9-di-methylpentadecane was evaluated by synthetic-baited pheromone traps or the level of damage that the insect caused to the leaves. The results showed failure of the MD that may be attributed to a combination of several technical factors [107,115]. Dispenser types, like aerosol [116] or aerial applications [117], release points in the field and treatment period still need to be tested for CLM control.

### 5.5. Biopesticides

#### 5.5.1. Botanical Pesticides

Biopesticides are bioproducts based on plant sources presenting remarkable advantages over conventional synthetic chemical pesticides, including: lower persistence in the environment, lesser phytotoxicity, more effectiveness, higher specificity toward target organisms, reduced pest management costs, and a low toxicological and ecotoxicological risk for field workers, consumers and the environment [118].

Studies on the efficacy and the use of botanical pesticides has been largely reported in the literature, and even more, recommended by international organizations as a more sustainable manner to control pests) [19,119,120,121,122,123]. Raw vegetal materials for biopesticide development are obtained from barks, leaves, roots, flowers, fruits, seeds, cloves, rhizomes, and stems of plants belonging to several botanical families. The derived substances from the materials processing are generally plant extracts, essential oils, or both [124]. Commercialized pesticides from plants, such as pyrethrum, neem and sabadilla, are some examples of biopesticides of the least toxicity to non-targets organisms, such as pollinators and fish [125].

Botanical aqueous extracts of (*Toona ciliata*, *Trichilia casaretti*, *Trichilia pallida*, *Trichilia catigua*, *Chenopodium ambrosioides,* and *Azadiracta indica*) have been evaluated against CLM eggs, larvae, and pupae under laboratory conditions. According to the results, the aqueous extract of *C. ambrosioides* and *T. casaretti* killed 50% of eggs against 45% for *T. ciliata*. Moreover, pupae *treated with A. indica* extract showed the highest level of mortality (100%) followed by *T. pallida* 75% and *C. Ambrosioides* 62%. In relation to larvae mortality, *A. indica* and *T. pallida* extracts were the most effective, killing 70% against 50% observed for *C. ambrosioides* [126]. These results might be useful in IPM for coffee leaf miner insects (*L. coffeella*). Activity against CLM larvae was reported by soaking treatment with extracts from *Achillea millefolium*, *Citrus limon*, *Glechoma hederacea*, *Malva sylvestris*, *Mangifera indica*, *Mentha spicata*, *Mirabilis jalapa*, *Musa sapientum*, *Ocimum basiculum*, *Petiveria alliaceae*, *Porophyllum ruderale*, *Psidium guajava*, *Rosmarinus officinalis*, *Roupala montana*, *Sambucus nigra,* and *Tropaeolum majus* [127]. The extracts of *Plantago lanceolata* and *Momordica charantiaplants* reduced oviposition and egg hatching, and fecundity for females obtained from eggs treated with the *M. charantia* [128].

Combining biopesticides and nanoscale-based delivery methods is now being explored to increase efficacy while limiting the negative impacts traditionally seen through the use of pest control means [129]. Nanotechnology offers the advantages of using nanomaterials presenting novel and enhanced features compared to bulk materials. The remarkable physicochemical properties of these materials generate applications in agriculture as pesticides and platforms for gene delivery [130,131].

#### 5.5.2. Nano-Biopesticides

Nano-biopesticides constitute nanoencapsulated (or nanoentrapped) pesticides, which can be bioactive compounds (biopesticides) and/or agrochemicals (e.g., insecticides), capable of controlling and inhibiting the growth of plant insect pests. Thus, nano-biopesticides comprise the encapsulation and/or entrapment of biopesticides, which are obtained from bacteria, fungi, plants, or animals (e.g., plant extracts and essential oils, fungal and bacterial biomolecules) [132]. The encapsulation not only optimizes stability, solubility, permeability, and specificity of pesticides, but also promotes a sustained release of them [133].

The agricultural nanoformulations are commonly based on metallic nanoparticles, polymeric nanoparticles, nanoemulsions, lipid nanoparticles, or carbon-based nanostructures. Silver nanoparticles (AgNPs) synthesized using the leaf extract of *Annona reticulata*, and the AgNPs showed insecticidal activity against *Sitophilus oryzae*, an insect that damages rice grains [134]. Nanoemulsions produced with *Pimpinella anisum* essential oil presented activity against the red flour beetle (*Tribolium castaneum*), a stored grain pest [135]. Likewise, solid lipid nanoparticles produced with geranium essential oil (*Pelargonium graveolens*) were reported as a control agent of black cutworm *Agrotis ipsilon* [136]. Similarly, graphene oxide nanocomposites loaded with pesticides (pyridaben, chlorpyrifos, and beta-cyfluthrin) enhanced acaricidal activity against spider mite [137].

## 6. Conclusions

There is great demand for control products of this pest that are less toxic, highly specific, with less impact on the population of natural enemies, and that result in lower production costs. Some biotechnological alternatives can generate products to meet the demand for sustainable, durable, and safe solutions for the specific control of this pest, besides reaching the most demanding markets, with approaches closer to holistic pest management (HPM) [19,138,139].

Development of coffee leaf miner resistant/tolerant cultivars remains a strong tendency. The current breeding programs began with *C. arabica* and *C. racemosa* crossings. Individuals belonging to the offspring of the second round of natural backcrosses (RC2) were hybridized with *C. arabica* commercial cultivars and generated new registered cultivars [77,140]. However, further investigation concerning the molecular basis of the resistance introgressed in *C. arabica* cultivars is required to keep the high-performance traits of grain yield and quality of the new genotypes.

CRISPR/Cas9 technology could circumvent some traditional breeding limitations to develop cultivars resistant to the CLM. Gene editing could provide both precise genome modifications and attenuation of regulatory restrictions on genetically engineered crops [141]. Despite the controversy surrounding GMOs in the agricultural sector, coffee is one of the very few woody species that has a validated protocol to mutate *C. canephora* with CRISPR/Cas9 A [91].

Biorational pesticides strategies to control *L. coffeella* must consider the important role of parasitoids, predators, and insect pathogens on enhancing the natural mortality of the CLM in field. Albeit biological control alone presents limitations in the efficiency and durability of treatments, it is positive in integrated control systems, particularly in organic farming.

Promising results using plant extracts against CLM encourage the research of improved biopesticides to be integrated in more robust and sustainable pest management systems. Despite the lack of scientific papers describing the use of agricultural nanoformulations to control major coffee pests like the CLM, these recent advances to control pathogens and pests in other crops strongly suggest this possibility in the forthcoming years.

## Data Availability

Not applicable.

## Supplementary Materials

The following are available online at https://www.mdpi.com/article/10.3390/insects12121130/s1, Table S1: Active compounds currently available and registered at the Brazilian Ministry of Agriculture, Livestock and Supply (MAPA).

## Appendix A

**Table A1:** Main hymenopteran species found in coffee growing areas parasitizing *Leucoptera* sp.

Family	Species	Country (a) % Parasitism (b)	Reference
Braconidae	*Apanteles bordagei*	(a) Tanzania (b) 20–75%	[94]
Encyrtidae	*Ageniaspis* sp.		
Eulophidae	*Cirrospilus variegatus* *Closterocerus ritchiei (sin. Achrysocharis ritchiei)* *Elasmus leucopterae* *Pediobius coffeicola*		
Braconidae Encyrtidae	*Apanteles bordagei* *Parahormius* sp. *Ageniaspis* sp.	(a) Kenia (b) 17–32%	[99]
Eulophidae	*Closterocerus ritchiei (sin. Achrysocharis ritchiei)* *Zagrammosoma variegatum* *Pediobius coffeicola* *Elasmus* sp. *Chrysonotomyia* sp.		
Braconidae	*Mirax insulatris*	(a) Puerto Rico (b) 19.5–23.5%	[95]
Eulophidae	*Achrysocharoides* sp. *Zagrammosoma* spp. *Cirrospiloideus* sp. *Horismenus* spp. *Chrysonotomyia* sp.		
Braconidae	*Centistidea striata* *Orgilus niger* *Stiropius reticulatus* *Mirax* sp.	(a) Brazil (b) 8–44%	[28,92,97]
Eulophidae	*Closterocerus coffeellae* *Cirrospilus* sp. *Horismenus* sp. *Neochrysocharis coffeae* *Proacrias coffeae* *Tetrastichus* sp.		
Braconidae	*Allobracon* sp. *Stiropius letifer*	(a) Mexico (b) ≤10%	[96]
Eulophidae	*Cirrospilus* spp. *Closterocerus* spp. *Elachertus* spp. *Horismenus* spp. *Miotropis* sp. *Neochrysocharis* spp. *Pnigalio* spp. *Zagrammosoma* spp.		
Eulophidae	*Zagrammosoma multilineatum* *Pnigalio sarasolai* *Closterocerus* spp. *Horismenus* sp. *Apleurotropis* sp.	(a) Colombia (b) 58–89%	[10]
